# Clinical Trials of Focused Ultrasound for Brain Tumors

**DOI:** 10.3390/cancers17030513

**Published:** 2025-02-04

**Authors:** Victor M. Lu, Toba N. Niazi

**Affiliations:** 1 Department of Neurological Surgery, University of Miami Miller School of Medicine, Jackson Health System, Miami, FL 33136, USA; 2 Department of Neurological Surgery, Nicklaus Children’s Hospital, Miami, FL 33155, USA

**Keywords:** focused ultrasound, FUS, brain tumor, DIPG, glioblastoma, clinical trial

## Abstract

Background: It is unclear as to where we stand with respect to utilizing emerging focused ultrasound (FUS) technology in the setting of brain tumor treatment in pediatric patients, such as malignant diffuse intrinsic pontine glioma, and various adult counterparts. Correspondingly, the aim of this study was to present a contemporary summary of all pertinent clinical trials to date. Methods: The ClinicalTrials.gov database was reviewed in January 2025 for all possible clinical trials involving FUS in the management of brain tumors. These were then screened against selection criteria to identify pertinent clinical trials. Results: A total of 30 clinical trials were identified. The majority were focused on adult patients (24/30, 80%), with the most common tumor type being glioblastoma (GBM) (14/30, 47%). There were also trials focused on pediatric patients only (5/30, 17%), as well as diffuse intrinsic pontine glioma (DIPG) (5/30, 17%). The most prevalent primary outcome of interest was safety (26/30, 87%). The majority of trials were active, either recruiting currently (12/30, 40%), or active but not recruiting currently (3/30, 10%). North America (22/30, 73%) was the most common location for the primary coordinating institution, and the median number of institutions per trial was one. The median expected start year for all trials was 2021, and the completion year was 2024. To date, there have been no results (interim or final) formally reported, although preliminary reports in the literature indicate this to be a safe procedure. Anecdotal trends suggest later trials target the blood-brain barrier more, involve more pediatric patients, and are more based in the United States. Conclusion: There exists a number of early-stage clinical trials investigating FUS to treat a variety of brain tumors in pediatric patients, as well as adult patients, with a significant clinical potential to improve outcomes. To date, no official results have been published, however anecdotal evidence is promising, and a number of results are expected soon.

## Simple Summary

There are malignant brain tumors in pediatric and adult patients that currently have no innovative treatment approaches to improve outcomes. Focused ultrasound (FUS) is an emerging treatment modality that can both target brain tumors and also assist in other treatments reaching the tumor by overcoming the blood-brain barrier (BBB). However, it is unclear how many clinical trials are underway investigating the use of FUS to treat these tumors. Therefore, we summarized all trials registered in the ClinicalTrials.gov database that are investigating FUS in the management of brain tumors. We found a total of 30 early-stage clinical trials investigating FUS to treat a variety of brain tumors in pediatric patients, as well as adult patients, with a significant clinical potential to improve outcomes. To date, no official results have been published, however anecdotal evidence is promising, and a number of results are expected soon.

## 1. Introduction

Brain tumors remain prevalent amongst adult and pediatric patients, with an estimated 94,000 new cases estimated to be diagnosed in the United States this year alone [1]. Of these, approximately one quarter will be malignant in nature which will portend to a median five-year survival rate of 35%. There are many reasons as to why these malignant tumors portend to such a dismal prognosis. In pediatric patients, diffuse intrinsic pontine glioma (DIPG) resides primarily in the brainstem which is surgically a very difficult place to access [2], and is relatively protected from systematic therapies due to the blood-brain barrier (BBB) [3]. In adults, despite surgical resection, glioblastoma (GBM) remains refractory to many of the therapies currently available [4].

Focused ultrasound (FUS) is an emerging technology that may prove beneficial in treating brain cancers in two different ways. It utilizes ultrasonic waves of different wavelengths to target lesions and anatomy in a spatially coordinated manner, minimizing damage to tissue outside the target area [5,6]. High-intensity FUS provides an ablative intervention that can be used to primarily target and eliminate tumor cells [7]. Similar to radiation treatment, direct targeted ablation can be cytotoxic to brain tumor cells and FUS has already proven successful in treating epileptogenic brain lesions [8]. The benefit of FUS technology is that it can be very targeted, minimizing the risk of collateral damage which has historically limited the use of radiation treatments for brain tumors especially in pediatric patients [9]. Low-intensity FUS on the other hand provides a disruptive intervention, that has been shown to disrupt the BBB which can allow for improved therapy delivery to brain tumors [10,11]. The BBB has for a long time been seen as the barrier to the delivery of successful treatments for malignant brain tumors, and as such, FUS can serve as a modality to overcome this historic limitation [12].

For tumors outside the central nervous system, such as prostate and bone cancers, FUS has become a popular treatment option built upon promising clinical trials [13,14]. With respect to brain tumors, however, our understanding of how FUS can be used remains relatively nascent. There are a number of clinical trials that have been registered, however, progress to date with respect to in-human clinical trials remains unclear. Correspondingly, the aim of this study was to present a contemporary summary of the progress all clinical trials have made to date.

## 2. Methods

### 2.1. Search Strategy

The search strategy was designed to capture all possible clinical trials with registration in the U.S. National Library of Medicine ClinicalTrials.gov registry (https://clinicaltrials.gov/, accessed on 1 January 2025) investigating an FUS as a therapy to treat brain tumors. ClinicalTrials.gov is a database of privately and publicly funded clinical trials on a wide range of diseases and conditions conducted around the world, providing information on over 300,000 research studies conducted across all states of the U.S. and in over 200 countries worldwide [15,16]. Databases were searched and screened in 1 January 2025 using the following string of search terms for condition or disease: ‘focused ultrasound’ and ‘brain tumor’. Synonyms used were ‘brain neoplasm’, ‘glioblastoma’, ‘glioma’, and ‘DIPG’.

### 2.2. Selection Criteria

For inclusion into our study, trials were required to investigate (1) a brain tumor cohort with (2) FUS used in the management of the disease. There was no limitation on brain tumor histology. Trials involving other neurologic diseases were not included. There were no limitations set on age, nature of tumor, or geographic location. Trials were deemed to treat ‘general tumors’ when their inclusion criteria did not discriminate between malignant or benign tumors, primary, or recurrent tumors, histology, pathology, or location.

### 2.3. Data Outcomes

The following outcomes were then extracted from the database: National Clinical Trial (NCT) number, title, primary institution, country of origin of primary institution, number of institutions involved, age group of enrolment, brain tumor type, recruitment status, type of primary intervention, primary outcome, availability of results, FUS type, use of any adjuvant therapies, funding status, (expected) start and completion year, and enrolment target [17]. All data analyses, including the generation of figures and tables, were performed using STATA 14.1 (StataCorp, College Station, TX, USA).

## 3. Results

### 3.1. Search Strategy

The initial search yielded 31 trials for screening. One trial was excluded because the neurological disease of interest was not a tumor. Ultimately, 30 trials were identified as appropriate for review in this study. Summary parameters are summarized in Table 1. Select individual data are listed in Table 2.

### 3.2. Trial Focus

**Figure 1:**
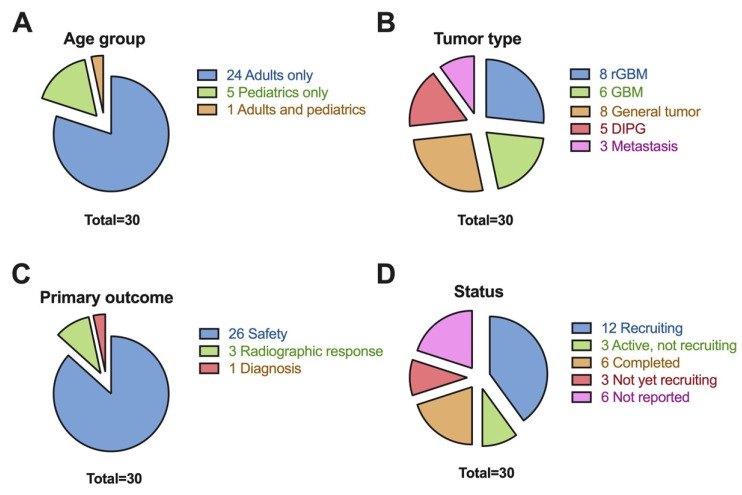
Trial status parameters of (**A**). age group, (**B**). tumor type, (**C**). primary outcome, and (**D**). trial status, across all 25 studies. rGBM, recurrent GBM; GBM, glioblastoma; DIPG, diffuse intrinsic pontine glioma.

The majority of the trials were focused on adult patients (24/30, 80%), with the remainder being pediatric patients only (5/30, 17%), and one (1/30, 3%) involving all ages (Figure 1A). With respect to brain tumor histology, the most common was GBM (13/30, 43%) which consisted of both recurrent GBM (8/30, 27%) and primary GBM (6/30, 20%) (Figure 1B). DIPG (5/30, 17%) was also common within trials involving pediatric patients, and brain metastatic disease (3/30, 10%) was the other category of brain tumor seen amongst these clinical trials. In terms of brain tumor categories, however, the most common was general brain tumors (8/30, 27%).

### 3.3. Trial Outcomes

For primary outcomes, the most common was safety (26/30, 87%), evaluating adverse events during FUS treatment (Figure 1C). Other primary outcomes included radiographic response (3/30, 10%), and then diagnosis (1/30, 3%). A total of 21 (70%) trials also reported secondary outcomes—the most common being radiographic response (12/21, 57%) followed by clinical performance (6/21, 29%), and safety (3/21, 14%).

### 3.4. Trial Status

In terms of trial status (Figure 1D), the majority of trials were active and recruiting (12/30, 40%), with some others active but not recruiting (3/30, 10%). The remainder of the trials were completed (6/30, 20%), not yet recruiting (3/30, 10%), or with an unknown non-updated status (6/30, 20%). No results (interim or final) have been officially reported yet for any of these trials at the time of publication.

### 3.5. Trial Treatment

**Figure 2:**
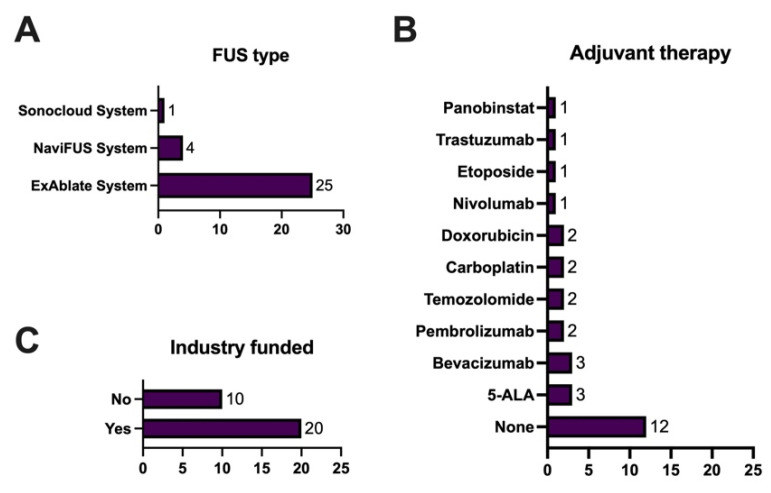
Trial treatment parameters of (**A**). focused ultrasound (FUS) type, (**B**). adjuvant therapy use, (**C**). industry funding status, across all 30 studies.

The most common FUS device used was the Exblate system (25/30 trials, 83%), followed by the NaviFUS system (4/30, 13%) and Brainsonix Sonocloud (1/30, 3%) system (Figure 2A). The anatomical target of the FUS was the BBB in the majority of trials (20/30, 67%) with the tumor lesion the other anatomical target in the remaining trials (10/30, 33%). There were 18 (60%) trials that utilized concurrent chemotherapy with FUS (Figure 2B). The most common agents reported were 5-ALA and bevacizumab (3/30 each, 10%), and then pembrolizumab, temozolomide (Stupp protocol), carboplatin and doxorubicin (all 2/30, 7%). Finally, the majority of these trials (20/30, 67%) reported industry funding (Figure 2C).

### 3.6. Trial Logistics

**Figure 3:**
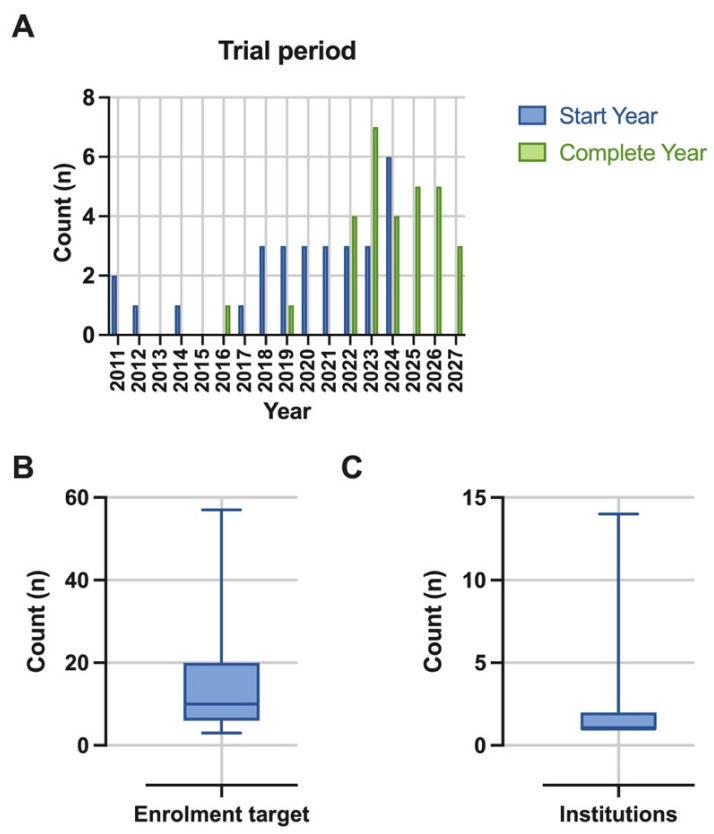
Trial logistic parameters of (**A**). expected start and completion years, (**B**). enrolment target, (**C**). number of institutions involved (plotted as box-and-whisker plot of median and range), across all 30 studies.

**Figure 4:**
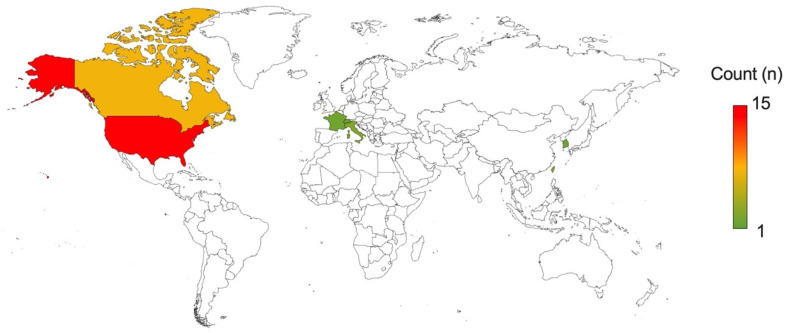
Geographic distribution of primary coordinating trial sites for all 30 trials.

The median start year for these trials was 2021 (range, 2011–2024) (Figure 3A). The median completion year for these trials is 2024 (range, 2016–2027). The median target enrolment was 10 patients (range, 3–57) (Figure 3B). The primary corresponding institution was located in seven different countries (Figure 4)—most commonly in the United States (15/30, 50%). The other location in North America was Canada (7/30, 23%). In Asia, trials were coordinated in Taiwan (3/30, 10%), and South Korea (1/30, 3%). The remainder of the trials were coordinated in Europe, in Italy (2/30, 7%), and then France and Switzerland (both 1/30, 3%). The median number of institutions involved was one institution (range, 1–14), with four (13%) trials involving sites in more than one country (Figure 3C).

## 4. Discussion

The use of FUS in treating brain tumors is an emerging possibility, evidenced by the number of active clinical trials that have arisen in recent years. We demonstrate that a number of these trials are currently active and are mostly based in North America. Although the most common target demographic is malignant brain tumors in adults, there are a number of pediatric-based trials investigating malignant brainstem tumors as well. Given its infancy, the safety of FUS technology is the most common primary outcome, with a number of further trials also studying clinical response.

Amongst the FUS clinical trials, all age groups were considered as well as a number of different brain tumor pathologies. The most common combination was recurrent GBM in adult patients, a diagnosis known to confer poor prognosis for which experimental therapies have become common [18]. Furthermore, a number of trials focused on pediatric brainstem tumors. The DIPG pathology is notoriously known to be resistant to standard adjuvant therapies [19]. The use of FUS in this setting reflects the hypothesis that overcoming the BBB can provide therapeutic benefit to this similarly dismal prognosis [11]. However, this technology is not limited to malignant brain tumors based on current trials, as a number of other pathologies including general tumors and brain metastases are also under investigation in the clinical trials.

This review demonstrates that the investigation of FUS to treat brain tumors remains relatively novel, with no clinical trial to date having disclosed or reported any results officially. This compares to multiple other tumor types that FUS has been approved for use by the Food and Drug Administration (FDA) within the United States [20]. Indeed, there are only a handful of trials that are listed as complete at the time of review. The median year of expected trial completion is 2024, as such we stand on the precipice of many of trials being completed. The only surrogate for safety that can be inferred at this time is that no trial has been suspended or withdrawn to date.

Although no clinical trial has officially reported its results, there are a number of published preliminary findings to consider. First, at our own institution, we have published our initial experience with treating benign intracranial tumors in pediatric patients with high-intensity FUS, primarily that of hypothalamic hamartoma (NCT03028246) [8]. The authors showed that hat FUS thermoablation of centrally located brain lesions in adolescents and young adults can be performed safely and that it provides therapeutic benefits for associated symptoms. Next, a clinical trial (NCT05123534) investigating 5-ALA analog SONALA-001 concurrently with low-intensity FUS in pediatric DIPG reported that their first patient tolerated the procedure well without any adverse effects [21]. An MRI was performed post-treatment day 1 and demonstrated no adverse changes, such as edema or hemorrhage, within the sonicated areas. Another trial (NCT03712293) investigating adult GBM outcomes following FUS with temozolomide in six patients showed the survival rate up to 1 year was 100%, and only two of the patients were found to have a recurrence of the disease [22]. Promisingly, none of the six patients had immediate or delayed BBB-related complications [23].

What the ideal FUS treatment regimen looks like in the future is not clear and is likely dependent on pathology. Approximately just under half of the trials incorporate FUS with concurrent or adjuvant chemotherapy, the most common being familiar agents to the current standard of care. Given one of the primary effects of FUS is BBB disruption, the concurrent use with chemotherapy is intuitive in the sense of maximizing tumor penetration [24]. The other half of the trials we report utilize FUS for its therapeutic effect of ultrasonic ablation [25]. As such, adjuvant therapy was not necessarily a needed investigative component for these trials. Results of these trials will therefore dictate in the future whether FUS can be considered a stand-alone therapeutic option for brain tumors, or a complementary one. This is reflected in the majority of primary outcomes being patient safety at this point in time. Once safety is established, more advanced clinical trials can start to truly elucidate clinical benefit.

The breadth of indications for the currently listed clinical trials indicates likely the direction FUS technology is heading in the neuro-oncology sphere. Although the majority of cases were focused on the therapeutic benefit, be that by safety or radiologic response, it is significant to note that this technology is also being utilized from a diagnostic perspective. A clinical trial (NCT04940507) is sampling both blood and cerebrospinal fluid (CSF) for circulating tumor DNA (ctDNA) after FUS treatment to aid in diagnosing specific brain tumors. Liquid biopsies have been shown to be possible already in this setting of malignant brain tumors such as GBM [26] and DIPG [27], with the idea of FUS enhancing the sensitivity and specificity of diagnoses by enabling more ctDNA and other tumor materials to be secreted into circulation after the BBB is disrupted [28]. All in all, FUS in the setting of brain tumors has not only the potential for treatment but also to aid in non-invasive diagnoses which when conducted simultaneously, may bring FUS to the forefront of brain tumor management in the future [29].

The majority of the clinical trials currently registered in the ClinicalTrials.gov registry are being coordinated within North America and are funded by industry. This can in part be attributed to the primary FUS device used most in these trials being Exblate©, a product of InSightec Ltd. (Tirat Carmen, Israel), which has headquarters located in the United States. Nonetheless, it is encouraging to see a number of trials are multi-institutional and across multiple continents. Not only does this reflect a growing interest in this new technology, but also it will assist from a trial design perspective in accumulating significant trial cohort sizes sooner.

Anecdotally there appears to be a trend in these trials that can be seen across the starting years. There were more trials in later years that targeted the BBB, as well as utilized some adjuvant or concurrent chemotherapy to enhance therapeutic delivery. This suggests a shift in paradigm to utilize FUS for its disruptive nature rather than its ablative nature in the setting of brain tumors. Further, there are more pediatric-specific trials in the latter half of the trials included, as well as those being coordinated by centers within the United States specifically.

There are limitations to our review. The first is the heterogeneity in which FUS is being employed in these clinical trials—from age group to tumor type of institution-dependent FUS protocols. This highlights that as results are reported, generalization across all brain tumor practices is limited. Furthermore, the ClinicalTrials.gov registry is limited in inferring clinical trial progress and design, and as such, the expected completion time for these trials may represent only the earliest time we can expect results to be finalized. Avenues and mandates for regular trial updates could provide more contemporary insights into how FUS treatment is preliminarily performed. Finally, there is a current lack of reported results for these trials, particularly those listed as completed. Although the registry is able to maintain an active record of trials that involve FUS, it is the responsibility of the registering party to maintain an active presence to ensure that we are advancing the field rather than stalling the field if a particular approach or combination proves ineffective.

Going forward, once initial results validate the clinical place for further investigation of FUS for these brain tumors in pediatric and adult patients, the next direction for trials to investigate will be FUS parameters to optimize the effect. This includes the number of FUS sessions as well as the target of the FUS in the setting of BBB effect versus tumor effect versus both [30]. The way the registry is designed, this information is not available through the publicly available database, and therefore any implication of this based on current trial designs is not known.

## 5. Conclusions

There have been twenty-nine clinical trials registered with the ClinicalTrials.gov database investigating the use of FUS to treat brain tumors. They most commonly focus on FUS safety in the setting of pediatric and adult malignant brain tumors, as both a stand-alone therapy, and concurrent therapy. These indications demonstrate the potential clinical advantages of this therapy to be minimally invasive, which is favorable for pediatric patients, and also to be innovative as it can allow for therapies to cross the blood-brain barrier that classically has hindered treatments for malignant brain tumors to date. Although there has been no formal disclosure of results from any clinical trial so far, the anecdotal and preliminary results to date are promising correlating to the growth in industry interest in this technology. However, the potential long-term oncologic, systemic, neurologic, and cognitive consequences of this novel therapy remain unclear, and as such, further study into the long-term safety of this therapy is needed and ongoing in current trials. Future trials will also focus on modifying the FUS target and dosage to maximize the desired clinical impact.

## Figures and Tables

**Table 1:** Summary parameters of trial focus and status. Percentage of total cohort in parenthesis. GBM, glioblastoma; DIPG, diffuse intrinsic pontine glioma.

Parameter	Cohort (n = 30)
Age group	
Adult only	24 (80%)
Pediatric only	5 (17%)
Adult and pediatric	1 (3%)
Tumor type	
Glioblastoma	13 (43%)
Recurrent GBM	8 (27%)
Primary GBM	6 (20%)
DIPG	5 (17%)
General tumor	8 (27%)
Metastasis	3 (10%)
Primary outcome	
Safety	26 (87%)
Radiographic response	3 (10%)
Diagnosis	1 (3%)
Trial status	
Currently recruiting	12 (40%)
Active not recruiting	3 (10%)
Completed	6 (20%)
Not recruiting	3 (10%)
Unknown	6 (20%)

**Table 2:** Selected individual clinical trial data. FUS, focused ultrasound; DIPG, diffuse intrinsic pontine glioma; GBM, glioblastoma; rGBM, recurrent glioblastoma; TMZ, temozolomide.

Trial Number	NCT Number	Start Year	End Year	Primary Country	FUS Device	Status	Tumor Type	Age Group	Adjuvant Therapy	Primary Outcome	Enrolment Goal
1	NCT01473485	2011	2022	Canada	ExAblate	Unknown	General	Adult	None	Safety	10
2	NCT01698437	2011	2016	Switzerland	ExAblate	Completed	General	Adult	None	Imaging	3
3	NCT00147056	2012	2022	US	ExAblate	Unknown	General	Adult	None	Safety	10
4	NCT02343991	2014	2022	Canada	ExAblate	Unknown	General	Adult	None	Safety	10
5	NCT03028246	2017	2024	US	ExAblate	Recruiting	General	Pediatric	None	Safety	10
6	NCT03616860	2018	2023	Canada	ExAblate	Completed	GBM	Adult	TMZ	Safety	14
7	NCT03626896	2018	2019	Taiwan	NaviFUS	Completed	rGBM	Adult	None	Safety	6
8	NCT03712293	2018	2023	South Korea	ExAblate	Completed	GBM	Adult	TMZ	Safety	9
9	NCT04021420	2019	2023	France	Sonocloud	Unknown	Metastasis	Adult	Nivolumab	Safety	21
10	NCT03714243	2019	2022	Canada	ExAblate	Unknown	Metastasis	Adult	Trastuzumab	Safety	10
11	NCT03551249	2019	2023	US	ExAblate	Completed	GBM	Adult	None	Safety	20
12	NCT04446416	2020	2023	Taiwan	NaviFUS	Completed	rGBM	Adult	Bevacizumab	Safety	6
13	NCT04440358	2020	2024	Canada	ExAblate	Active, not recruiting	rGBM	Adult	Carboplatin	Safety	13
14	NCT04417088	2020	2024	US	ExAblate	Active, not recruiting	rGBM	Adult	Carboplatin	Safety	30
15	NCT04998864	2021	2023	Italy	ExAblate	Unknown	GBM	Adult	None	Safety	5
16	NCT04804709	2021	2025	US	ExAblate	Active, not recruiting	DIPG	Pediatric	Panobinostat	Safety	3
17	NCT04940507	2021	2026	Canada	ExAblate	Recruiting	General	Adult	None	Diagnosis	50
18	NCT05383872	2022	2025	US	ExAblate	Recruiting	GBM	Adult	None	Safety	57
19	NCT05317858	2022	2024	US	ExAblate	Recruiting	Metastasis	Adult	Pembrolizumab	Safety	20
20	NCT05123534	2022	2025	US	ExAblate	Recruiting	DIPG	Both	5-ALA	Safety	40
21	NCT04845919	2023	2023	Italy	ExAblate	Not yet recruiting	GBM	Adult	5-ALA	Imaging	5
22	NCT05615623	2023	2025	Canada	ExAblate	Recruiting	DIPG	Pediatric	Doxorubicin	Safety	3
23	NCT05755399	2023	2026	US	ExAblate	Recruiting	General	Adult	None	Imaging	15
24	NCT05630209	2023	2026	US	ExAblate	Recruiting	DIPG	Pediatric	Doxorubicin	Safety	10
25	NCT05762419	2023	2027	US	ExAblate	Recruiting	DIPG	Pediatric	Etoposide	Safety	10
26	NCT05733312	2024	2025	US	ExAblate	Recruiting	General	Adult	None	Safety	6
27	NCT05879120	2024	2026	US	ExAblate	Not yet recruiting	rGBM	Adult	Pembrolizumab	Safety	10
28	NCT06039709	2024	2026	US	NaviFUS	Recruiting	rGBM	Adult	5-ALA	Safety	11
29	NCT06329570	2024	2027	US	NaviFUS	Not yet recruiting	rGBM	Adult	Bevacizumab	Safety	10
30	NCT06496971	2024	2027	Taiwan	NaviFUS	Recruiting	rGBM	Adult	Bevacizumab	Safety	32

## Data Availability

The original contributions presented in this study are included in the article. Further inquiries can be directed to the corresponding author.
